# Pennsylvania State Dental Society

**Published:** 1884-11

**Authors:** W. H. Trueman


### PENNSYLVANIA STATE DENTAL SOCIETY.

SIXTEENTH ANNUAL MEETING, HELD AT WILKESBARRE, JULY 29, 30 AND 31, 1884.

REPORTED FOR THE INDEPENDENT PRACTITIONER BY W. H. TRUEMAN, D. D. S.

(Continued from page 572.)

Dr. Chas. J. Essig, of Philadelphia, read a paper upon "Dental Diagnosis." He remarked: Pain or discomfort in or around a tooth, while usually the first symptom of a deviation from the normal condition either of the pulp or the surrounding tissues, may be merely sympathetic, and the organ seemingly affected perfectly sound. The differential diagnosis of the cause of the trouble, therefore, often demands much care and intelligence. The causes of pain in and around the dental organs at present recognized are comparatively numerous, but are often so obscure as to baffle for a time all efforts to find their origin. Even in the apparently simple matter of differentiating between the pain of periostitis and the purely neuralgic pain of an exposed pulp, much difficulty may occasionally be experienced. This was illustrated in a recent case. The patient complained of great pain near an upper tooth on the right side, which she could not definitely locate. Placing her finger on the first molar she remarked: "It begins here and extends toward the temple." On examining the tooth in question I found it devitalized, and the cavities in it filled with gold after the most approved contour methods, as were several of the other teeth near to it. The work was beautifully done, and suggested that the roots had been properly treated. There were marks on the gum over the tooth indicating the previous existence of an abscess, but at the time of examination there was not the least tenderness on percussion, and no loosening or elongation. In response to questions the patient said that neither heat nor cold aggravated the pain, but the recumbent position made it much worse. The fact of this tooth being devitalized, the evidence of previous trouble, and the statement of the patient that the recumbent position made the face throb, induced me in the hurry of the moment to treat it as a case of incipient periostitis. She reported the next morning unrelieved.

I now made a more thorough examination, determined to find the cause of pain, and asked the following questions; the answers will suggest to you how little we can depend upon the patient's observations in many of these cases. My first question was: "Does the pain come on after eating? No, it may come on at any time. Does the presence of sugar or sweets cause it? No. The recumbent position aggravates it? Yes; I could not lie down at all last night. Are you sure cold does not cause it? Yes; I have just eaten water ice without discomfort." In order to test this, I took up a syringe and threw a jet of not very cold water equally upon the first molar and the second bicuspid. Instantly the patient suffered the most acute pain. Here then was the trouble. The second bicuspid contained a very large gold contour filling. The absence of redness, swelling, loosening, and elongation, indicated that the pain was confined to the pulp alone. The filling was removed and found to have been very securely anchored by a number of retaining pits, one of which penetrated to the pulp. A fine exploring instrument was passed into it, and on its removal a small amount of pus came out, followed by bleeding.

Even in simple cases, where the patient can definitely indicate the affected tooth, diagnostic care is still necessary to successful treatment, particularly where the previous history of the tooth is unknown.

We may also meet with complications of a much more puzzling character than those here described. Recently such a case presented. The mouth had received no attention for ten years. There was great pain in the left inferior second or third molar. It required no little care to arrive at the conclusion that the pulp in the wisdom tooth was slightly exposed and fully alive, and that the pulp in the second molar had long been dead, and that the violet-colored nodule which was easily seen projecting through an opening from the pulp chamber was gum tissue, and not the pulp. It may be remarked that the difference in sensation between the two might have settled the question, but so far as response to contact of the instrument went, the patient did not seem able to distinguish between them. It is needless to say that the hasty or careless use of arsenic at the time of the first examination might have been serious. Another case even more forcibly illustrates the difficulty of diagnosis. The patient suffered from severe pain, swelling and elongation of the left inferior first molar, and a small circumscribed abscess on the right superior canine. Neither of the teeth had ever been decayed, but both were affected by pyorrhœa alveolaris. Aware that small circumscribed abscesses often occur during the progress of that disease without the pulp being involved, and that the sinus or pocket which forms along the tooth may by its encroachment cause the death of the pulp, and the usual more diffused abscess, the important point to determine was, which condition had we to deal with, as the treatment would depend entirely on the condition found. According to the patient's account the molar had been sensitive to cold a long time, and had been so tender that it was practically useless. The canine had never given any trouble. I inferred that the pulp of the molar had died, and we had the usual form of alveolar abscess to deal with, and that the canine was merely affected by one of the peculiar circumscribed abscesses almost characteristic of pyorrhœa alveolaris. Application of ice-cold water confirmed the diagnosis. In the case of the molar an opening was made into the pulp chamber, and the abscess over the canine was opened and treated with chloride of zinc. The relief in each case was prompt and permanent.

It may be urged that the cases cited are somewhat unusual; let us grant at once that such is the case; I hold that it is in just such cases that difficulty, if not danger, is to be encountered, and therefore unusual cases demand extraordinary care.

*Dr. William H. Trueman*, of Philadelphia—Was very much interested in Dr. Essig's paper. We are constantly meeting with cases like those he had cited, cases where the diagnosis is alike very difficult and very important. The third case he mentioned was especially so; and how easy, how very easy in such a case to make a mistake without being at all careless, and under some circumstances such a mistake, as the doctor remarks, might be very serious. It seems very easy to distinguish a devitalized tooth from a vital one, and yet there are many instances in which he was unable to do so at the first examination; indeed in some cases, such as one that occurred some years ago with a tooth in his own mouth, it is almost impossible without actually opening into the tooth. The tooth referred to was repeatedly examined by several dentists of large experience, who were unable to decide as to its condition. About twelve years afterward it was opened into and found to be dead; probably had been so a long time, yet had never given any trouble, or shown the least sign of its true condition.

He was aware of the usual means of testing, and of the usual signs of death of the pulp; but we occasionally meet with cases where all signs and tests leave us in doubt. If we drill into the pulp supposing it dead, and find it alive, we have done irreparable injury. He would very much like to know how we can absolutely diagnose whether the pulp is dead or alive in these cases.

Then, again, we meet with other cases of difficult diagnosis, none more so perhaps than those where bony granules, pulp-stones, they are sometimes termed, are found in the pulp chamber. They may, and often do, cause intense pain, without its being localized sufficiently to enable us to detect the tooth in fault. Indeed, there may be more than one tooth in the same condition. At one time the pain may settle in a certain tooth; in a few hours it has passed from that and is felt in another, or the pain may be so diffused and general that the patient cannot locate it at all. He had such a case some time ago. It was only after repeated examinations that a lower molar gave a suspicion of tenderness on percussion; the case then passed to another dentist, who found the same tooth very slightly sensitive, and drilled into and devitalized it. Granules of bone were found in the pulp. The tooth was treated and filled with every possible care, yet a year after the patient complained that it was so sore and painful during mastication that he seriously contemplated having it extracted. This is too often the history of such cases, before it is possible to locate the seat of injury; the irritation has continued so long, or has been so severe, that we really have a neuralgic trouble very hard to subdue unless the tooth is extracted.

We may also refer to the trouble we have from nerve sympathy; for instance, an exposed pulp in the lower jaw giving rise to pain in an upper tooth, etc., the tooth really in fault giving no sign. This was illustrated in a recent case; severe pain in a lower first bicuspid, slightly decayed, was really due to an exposed pulp in the lower third molar of the same side. It was very hard to convince the patient that a tooth he had never felt, was not conscious of being at all sensitive, was probably responsible for the pain he suffered; yet its removal gave immediate relief. We might multiply these cases. Then we may have an abscess appearing far distant from the tooth causing it ; or periosteal irritation entirely due to mal-occlusion, where we would least look for it ; or dental trouble due entirely to nerve sympathy, the real irritation being far removed from the dental organs ; and many other unusual cases which your experience will suggest. True, they *are* unusual, some of them *very* unusual cases; but as Dr. Essig well says, they are cases requiring extreme care to accurately diagnose, and cases in which accurate diagnosis is very important.

The question was raised whether it was possible to have an alveolar abscess on a tooth with a living pulp. Dr. Darby related a case of abscess where he had no doubt the pulp was alive. Dr. Gerhart also stated that he met with a case where two living teeth were abscessed. Dr. Green stated that in 1848 he met with a canine discharging freely, apparently abscessed, but afterwards found the pus came from the antrum of Highmore. He considered nodulary dentine the most difficult to diagnose of any condition we have to treat, and questioned whether it was not better in many cases to extract at once. It was so often associated with a neuralgic condition, that the chance of making the tooth permanently comfortable seemed very slight.

*Dr. Darby*—Related a case of a maiden lady of about forty-five years of age, who had consulted him, complaining of severe pain in the teeth. He had examined them several times and found nothing wrong. Five or six others had also examined the mouth with the same result. She had also consulted a number of physicians, and had been under their treatment without relief. The last time she was in his office he found that all the teeth had been extracted, but the pain still continued. In consultation with her physician they both agreed that the trouble was due to uterine irritation, perhaps aggravated by dyspepsia, from which she had long suffered.

*Dr. Gerhart*—Suggested that catarrh, which he considered an American disease, was in many difficult cases the real cause of trouble. The irritation associated with it at times, he had no doubt, through nerve sympathy, caused severe pain in and around the teeth.

*Dr. C. S. Beck*—Said, that in diagnosing obscure cases we must look beyond the point where pain is felt. We must consider the physical condition of the patient, the time of life, the condition of life, and the social surroundings. Those who are reared in luxury, and indulge in high living, and make but little exertion, who really are not filling the part in life nature designed they should, we will generally find suffer more intensely from nerve troubles. It seems to be nature's way of evening things up.

*Dr. Guilford*—Called attention to a small electric light introduced, which seemed to be entirely practical for use in the mouth, and promised to be a valuable aid in minute examinations of the teeth. When held behind the teeth it seemed to make the parts transparent, and the condition of the pulp was very plainly shown. If alive it could not be seen; if congested a change of color would be noticed, while a dead pulp seemed quite opaque, and a filling extending into the root shows black. This is so plainly seen that the exact condition of the root can be known with certainty. He thought it would prove valuable also in detecting incipient decay on the approximal surfaces.

The subject was passed, and the session adjourned.

